# Potent anti‐angiogenesis and anti‐tumour activity of pegaptanib‐loaded tetrahedral DNA nanostructure

**DOI:** 10.1111/cpr.12662

**Published:** 2019-07-31

**Authors:** Xueping Xie, Yuxin Zhang, Wenjuan Ma, Xiaoru Shao, Yuxi Zhan, Chenchen Mao, Bofeng Zhu, Yi Zhou, Hu Zhao, Xiaoxiao Cai

**Affiliations:** ^1^ State Key Laboratory of Oral Diseases, National Clinical Research Center for Oral Diseases West China Hospital of Stomatology Sichuan University Chengdu China; ^2^ Key Laboratory of Shaanxi Province for Craniofacial Precision Medicine Research, College of Stomatology Xi’an Jiaotong University Xi’an China; ^3^ Clinical Research Center of Shaanxi Province for Dental and Maxillofacial Diseases, College of Stomatology Xi’an Jiaotong University Xi’an China; ^4^ Department of Forensic Genetics, School of Forensic Medicine Southern Medical University Guangzhou China; ^5^ College of Basic Medicine Chengdu University of Traditional Chinese Medicine Chengdu China; ^6^ Department of Restorative Sciences, College of Dentistry Texas A&M University Dallas Texas

**Keywords:** anti‐angiogenesis, anti‐tumour, pegaptanib, pegaptanib‐TDNs, VEGF

## Abstract

**Objectives:**

Pegaptanib might be a promising anti‐tumour drug targeting VEGF to inhibit tumour vascular endothelial cell proliferation. However, the poor biostability limited its application. In this study, we took tetrahedron DNA nanostructures (TDNs) as drug nanocarrier for pegaptanib to explore the potent anti‐angiogenesis and anti‐tumour activity of this drug delivery system.

**Materials and methods:**

The successful synthesis of TDNs and pegaptanib‐TDNs was determined by 8% polyacrylamide gel electrophoresis (PAGE), capillary electrophoresis and dynamic light scattering (DLS). The cytotoxicity of pegaptanib alone and pegaptanib‐TDNs on HUVECs and Cal27 was evaluated by the cell count kit‐8 (CCK‐8) assay. The effect of pegaptanib and pegaptanib‐TDNs on proliferation, migration and tube formation of HUVECs induced by VEGF was examined by CCK‐8 assay, wound healing assay and tubule formation experiment. The cell binding capacity and serum stability were detected by flow cytometry and PAGE, respectively.

**Results:**

Pegaptanib‐TDNs had stronger killing ability than pegaptanib alone, and the inhibiting effect was in a concentration‐dependent manner. What's more, pegaptanib‐loaded TDNs could effectively enhance the ability of pegaptanib to inhibit proliferation, migration and tube formation of HUVECs induced by VEGF. These might attribute to the stronger binding affinity to the cell membrane and greater serum stability of pegaptanib‐TDNs.

**Conclusions:**

These results suggested that pegaptanib‐TDNs might be a novel strategy to improve anti‐angiogenesis and anti‐tumour ability of pegaptanib.

## 1 INTRODUCTION

Aptamers are DNA or RNA oligonucleotides, which can be synthesized and have high affinity and specificity to a number of biochemical targets.^1^, ^2^, ^3^ Aptamers have many advantages over antibodies such as cell‐free chemically synthesis, high tissue penetration, non‐immunogenicity, adaptable modification, low cost and thermostable.^4^ Therefore, aptamers have attracted extensive attention in terms of targeted therapy.^5^ Pegaptanib is an RNA aptamer that is specific to VEGF165, a subgroup of the VEGF family. In December 2004, the US FDA approved pegaptanib for the treatment of all types of AMD.^2^ As we all know, tumour blood vessels play an important role in tumour growth, providing essential oxygen and nutrients for tumour metabolism and metastasis.^6^, ^7^ VEGF is a very important regulator of endothelial cell growth and survival.^8^ So inhibiting VEGF may be a viable way to treat cancer.^9^, ^10^ Therefore, pegaptanib might be a promising candidate for VEGF‐targeting drugs for cancer therapy. There are few studies on the anti‐tumour effect of pegaptanib.

Pegaptanib is delivered through the bloodstream to the site of the tumour, which is different from intravitreal injection.^11^ The relatively poor biostability in vivo, such as the susceptibility to nucleases and removed from the circulation rapidly limit the use of pegaptanib in cancer treatment.^12^ It is important to introduce an effective aptamer delivery system to improve the biostability and half‐time in vivo. 13, 14, 15, 16Heo et al generated an aptamer‐antibody hybrid complex by reacting an anti‐continine antibody with the continnine‐conjugated pegaptanib aptamer, which suggested a novel aptamer delivery system for pegaptanib.^5^

**Figure 1:**
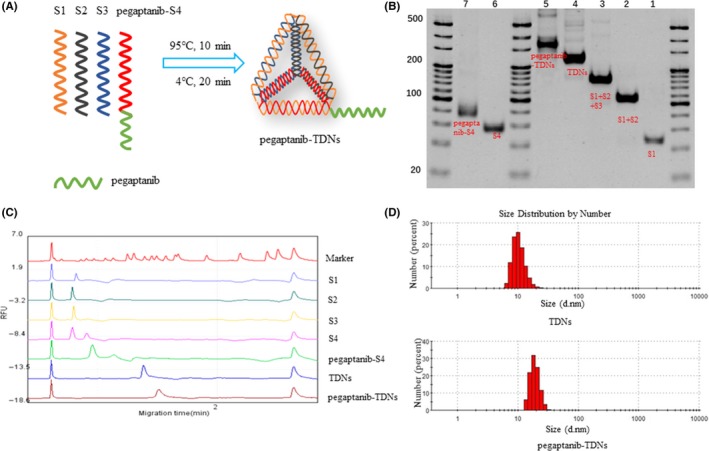
A, Sketch map of pegaptanib‐TDNs. B, Native PAGE to verify the assembly of TDNs and pegaptanib‐TDNs. C, The peak chart of marker and each molecule detected by capillary electrophoresis. D, Typical size distribution graphs of pegaptanib‐S4 and pegaptanib‐TDNs

DNA nanomaterials have attracted extensive attention in recent years due to their nanometer size, molecular recognition and controllability.^17^, ^18^, ^19^ TDNs, self‐assembled by four single‐stranded DNAs (ssDNAs) based on their highly specific Watson‐Crick base pairing, is one of the hot topics in the research field of the DNA nanomaterials.^20^, ^21^ In our previous study, we investigated the applications of TDNs in molecular regulation, disease therapy and drug delivery.^22^, ^23^, ^24^, ^25^, ^26^, ^27^ Zhang et al successfully transported antisense peptide nucleic acids (asPNAs) into methicillin‐resistant *Staphylococcus aureus* cells by TDNs to effectively inhibit bacterial.^26^ Hyukjin Lee et al showed TDNs could be regarded as siRNA nanocarrier to silence target genes in tumours.^28^ More interestingly, Ma et al synthesized an intelligent DNA nanorobot based on TDNs which enhance protein lysosomal degradation of HER2 in vitro.^29^ What's more, some chemotherapeutic drugs loaded TDNs could overcome drug‐resistant cancers.^27^, ^30^ In this study, we took TDNs as the nanocarrier of pegaptanib (Figure 1A) to investigate their effects of anti‐angiogenesis and anti‐tumour compared with pegaptanib alone.

## 2 MATERIALS AND METHODS

### 2.1 Synthesis of TDNs and Pegaptanib‐loaded TDNs (pegaptanib‐TDNs)

TDNs were prepared as previously reported.^31^ Four ssDNA strands in equal concentrations were mixed in TM buffer (10 mmol/L Tris‐HCl, 50 mmol/L MgCl2·6H2O, pH 8.0). The solution was heated to 95°C for 10 minutes and then cooled down to 4°C for 20 minutes. The pegaptanib‐TDNs were synthesized using S1, S2, S3 and pegaptanib‐S4 under the same conditions as above.

### 2.2 Characterization of TDNs and Pegaptanib‐TDNs

The successful synthesis of TDNs and pegaptanib‐TDNs was examined by 8% polyacrylamide gel electrophoresis (PAGE) and capillary electrophoresis.^32^, ^33^, ^34^ Capillary electrophoresis was directed by Qsep100^TM^ automatic nucleic acid protein analysis system. The hydrodynamic sizes of TDNs and pegaptanib‐TDNs were measured by A Zetasizer Nano‐ZS (Malvern Instruments).

### 2.3 Cell culture

HUVECs and Cal27 were purchased from the American Type Culture Collection (ATCC®CRL‐1730™, ATCC, USA; ATCC CRL‐2095). They were cultured in high glucose Dulbecco's modified Eagle's medium (DMEM) supplemented with 10% (*v*/*v*) foetal bovine serum (FBS) and 1% (*v*/*v*) penicillin/streptomycin antibiotics and maintained at 37°C in 5% CO_2_.

### 2.4 Cell proliferation assay

HUVECs and Cal27 were cultured in 96‐well microtitre plates at a density of 8 × 10^3^ cells/well. On the other day, 250 nmol/L and 375 nmol/L TDNs, pegaptanib and pegaptanib‐TDNs were added into the cell media, respectively. After incubation for 48 hours, cells were rinsed thrice with phosphate‐buffered saline (PBS), and the cell viability was measured by CCK‐8 assays. To detect the effect of these drugs on proliferation of HUVECs induced by VEGF, HUVECs were cultured in VEGF (25 ng/mL), VEGF (25 ng/mL) +TDNs (250 nmol/L or 375 nmol/L), VEGF (25 ng/mL) +pegaptanib (250 nmol/L or 375 nmol/L) and VEGF (25 ng/mL) +pegaptanib‐TDNs (250 nmol/L or 375 nmol/L). After 48 hours, the cell viability was monitored by CCK‐8.

### 2.5 Wound healing assay

This section was prepared on the basis of the previously reported methods.^35^, ^36^ HUVECs were seeded in 6‐well plates and cultured for 24 hours. After serum‐free starvation overnight, we used the sterilizer tip to scrape a two‐way wound at the bottom and washed the cells three times with PBS. The cells were treated with VEGF (25 ng/mL), VEGF (25ng/mL) +TDNs (375 nmol/L), VEGF (25 ng/mL) +pegaptanib (375 nmol/L) and VEGF (25 ng/mL) +pegaptanib‐TDNs (375 nmol/L). Wound closure was imaged after cultivation for 0 and 24 hours, respectively.

### 2.6 Measurement of the tube formation of HUVECs

50 μL Matrigel solution was added to each well of a 96‐well plate and incubated for 1 hour at 37°C. After being serum‐starved overnight, HUVECs were trypsinized and resuspended in high glucose DMEM with 0.5% FBS at 1 × 10^5^ cells/mL. 100 μL of the resuspended HUVECs was added to the Matrigel‐coated wells and incubated for 30 minutes to allow cell attachment. Subsequently, the media were replaced with high glucose DMEM containing VEGF (25 ng/mL), VEGF (25 ng/mL) +TDNs (375 nmol/L), VEGF (25 ng/mL) +pegaptanib (375 nmol/L) and VEGF (25 ng/mL) +pegaptanib‐TDNs (375 nmol/L). The cells were incubated at 37°C in 5% CO_2_ for 10 hours. After incubation, HUVECs were imaged by an inverted fluorescence microscope (Olympus IX73). The mean tube length was analysed by ImageJ.

### 2.7 Cell binding capacity of pegaptanib and pegaptanib‐TDNs to HUVECs

HUVECs were seeded in 6‐well plates. After 24 hours, pegaptanib‐cy5 (pegaptanib concentration: 0, 5, 10, 50, 100, 250 nmol/L) and pegaptanib‐TDNs‐cy5 (pegaptanib‐TDNs concentration: 0, 5, 10, 50, 100, 250 nmol/L) were added to the culture media. Cells were cultured for another 2 hours. Then, the cells were rinsed three times with PBS and digested with trypsin. The detached cells were collected into centrifuge tubes and centrifuged at 350 *g* for 5 minutes. The cell pellets were resuspended into 500 μL PBS. Subsequently, cell suspensions were measured by flow cytometry.

### 2.8 Detection of Serum Stability of Pegaptanib and Pegaptanib‐TDNs

Pegaptanib and pegaptanib‐TDNs were suspended in the high glucose DMEM with 10% (*v*/*v*) FBS and incubate at 37°C in 5% CO_2_ for 0, 2, 6, 8, 10, 12, 24, 36 hours, respectively. 8% PAGE was used to detect the degradation of pegaptanib and pegaptanib‐TDNs at different time points.

### 2.9 Statistical analysis

One‐way ANOVA (analysis of variance) or Student‐Newman‐Keuls test was used to perform statistical analysis of data and *P* < 0.05 indicated that group means were significantly different. All quantitative results were presented as mean ± standard deviation (SD).

## 3 RESULTS

### 3.1 Characterization of TDNs and pegaptanib‐TDNs

Pegaptanib was linked to the 5’ terminal of S4 to form pegaptanib‐S4. The ssDNA sequences were listed in Table 1. After the synthesis process, four single‐stranded nucleic acids were self‐assembled. 8% PAGE was applied to examine the successful synthesis of TDNs and pegaptanib‐TDNs. In Figure 1B, lane 1‐7 represented S1, S1 + S2, S1 + S2+S3, TDNs, pegaptanib‐TDNs, S4 and pegaptanib‐S4, respectively. Pegaptanib added to S4 and TDNs resulted in that pegaptanib‐S4, and pegaptanib‐TDNs migrated more slowly than S4 and pegaptanib‐TDNs. Capillary electrophoresis was also utilized to examine the synthesis of these materials. As shown in Figure 1C, the peak of pegaptanib‐S4 and pegaptanib‐TDNs shifted to the right compared with S4 and TDNs. Size of TDNs and pegaptanib‐TDNs which, respectively, was about 10nm and 22nm was measured by DLS (Figure 1D). All the results proved that pegaptanib was successfully loaded onto TDNs.

**Table 1:** Sequence of each single‐stranded nucleic acid

ssDNA	Sequence
S1	5´‐ATTTATCACCCGCCATAGTAGACGTATCACCAGGCAGTTGAGACGAACATTCCTAAGTCTGAA‐3´
S2	5´‐ACATGCGAGGGTCCAATACCGACGATTACAGCTTGCTACACGATTCAGACTTAGGAATGTTCG‐3´
S3	5´‐ACTACTATGGCGGGTGATAAAACGTGTAGCAAGCTGTAATCGACGGGAAGAGCATGCCCATCC‐3´
S4	5´‐ACGGTATTGGACCCTCGCATGACTCAACTGCCTGGTGATACGAGGATGGGCATGCTCTTCCCG‐3´
pegaptanib	5´‐C^G*G*AAU^C^A*G*U^G*A*A*U^G*C^U^U^A*U^A*C^A*U^C^C^G*‐3´‐dT‐5´
Pegaptanib‐S4	5´‐C^G*G*AAU^C^A*G*U^G*A*A*U^G*C^U^U^A*U^A*C^A*U^C^C^G*‐3´‐dT‐5´‐TTTTTACGGTATTG GACCCTCGCATGACTCAACTGCCTGGTGATACGAGGATGGGCATGCTCTTCCCG‐3´

*, 2´‐O‐methylated purines; ^, 2´‐fluorine‐modified pyrimidines.

### 3.2 Cell proliferation of HUVECs and Cal27

**Figure 2:**
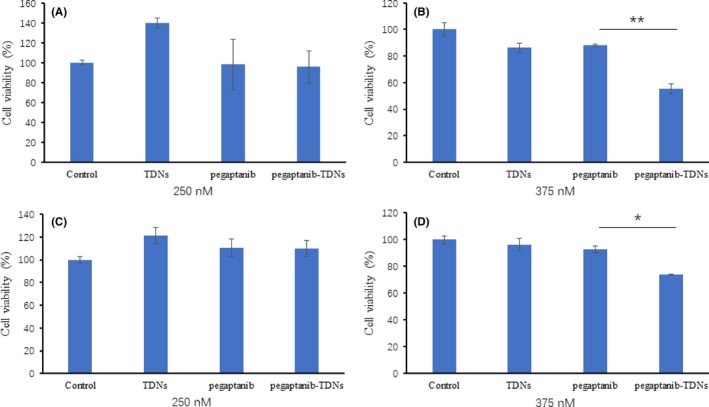
Cell proliferation of HUVEC exposed to TDNs, pegaptanib and pegaptanib‐TDNs. A, 250 nmol/L, (B) 375 nmol/L. Cell proliferation of Cal27 exposed to TDNs, pegaptanib and pegaptanib‐TDNs. C, 250 nmol/L, (D) 375 nmol/L. Data are presented as mean ± SD (n = 3). Statistical analysis: *P < 0.05, **P < 0.01

The cell proliferation was evaluated by CCK‐8 assay. As shown in Figure 2A, when HUVECs were incubated in the 250 nmol/L materials, cell proliferation had not been influenced by TDNs, pegaptanib and pegaptanib‐TDNs. When the materials were at the concentration of 375 nmol/L, TDNs and pegaptanib had no effect on the proliferation of HUVECs, whereas 45% of HUVECs were inhibited by pegaptanib‐TDNs (Figure 2B). In order to investigate the effect of these materials on the proliferation of Cal27, the human oral cancer cells, in the same way, the cells were cultured in the materials at 250 and 375 nmol/L concentrations, respectively. None of them at 250 nmol/L had a significant effect on the proliferation of Cal27 (Figure 2C). When the concentration went up to 375 nmol/L, 26% of Cal 27 were killed by pegaptanib‐TDNs, and meanwhile, both TDNs and pegaptanib had no toxic effect on the viability of Cal27 (Figure 2D). The inhibiting effect of pegaptanib‐TDNs on the proliferation of HUVECs and Cal27 was in a concentration‐dependent manner.

### 3.3 Pegaptanib‐TDNs can inhibit proliferation, migration and tube formation of HUVECs induced by VEGF

**Figure 3:**
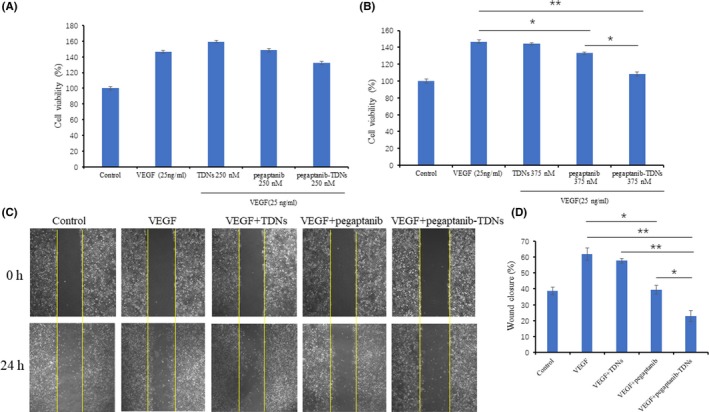
Cell proliferation of HUVECs induced by VEGF was inhibited by pegaptanib‐TDNs in a dose‐dependent manner; (A) 250 nmol/L, (B) 375 nmol/L. C, Pegaptanib‐TDNs inhibited the VEGF‐induced migration of HUVECs. The materials concentration was 375 nmol/L. D, The semi‐quantitative analysis of wound closure. Data are presented as mean ± SD (n = 3). Statistical analysis: *P < 0.05, **P < 0.01

VEGF, an angiogenic factor, is crucial to promote the proliferation of HUVECs. HUVECs were incubated with VEGF (25 ng/mL), VEGF (25 ng/mL) +TDNs (250 nmol/L or 375 nmol/L), VEGF (25 ng/mL) +pegaptanib (250 nmol/L or 375 nmol/L) and VEGF (25 ng/mL) +pegaptanib‐TDNs (250 nmol/L or 375 nmol/L) for 48 hours. In Figure 3A, pegaptanib (250 nmol/L) and pegaptanib‐TDNs (250 nmol/L) could not inhibit the proliferation of HUVECs induced by VEGF. In Figure 3B, 375 nmol/L pegaptanib‐TDNs had a stronger inhibitory effect compared with 375 nmol/L pegaptanib alone. Pegaptanib and pegaptanib‐TDNs reduced VEGF‐induced HUVECs proliferation in a dose‐dependent manner.

HUVECs migration is a necessary process of angiogenesis. VEGF is a chemokine of HUVECs, which can promote HUVECs migration by activating cytoskeleton remodelling signalling pathways.^9^, ^37^ A wound healing assay was applied to assess the inhibition of pegaptanib and pegaptanib‐TDNs on VEGF‐induced migration of HUVECs. After incubation with 375 nmol/L pegaptanib for 24 hours, wound closure was 39%, whereas it was just 23% when cell cultured in 375 nmol/L pegaptanib‐TDNs for 24 hours. At the same time, wound closure in control and VEGF group was 38% and 62% (Figure 3C,D). Both pegaptanib and pegaptanib‐TDNs could inhibit migration of HUVECs induced by VEGF. But pegaptanib‐TDNs showed remarkably stronger inhibition compared with pegaptanib alone which was statistically significant.

**Figure 4:**
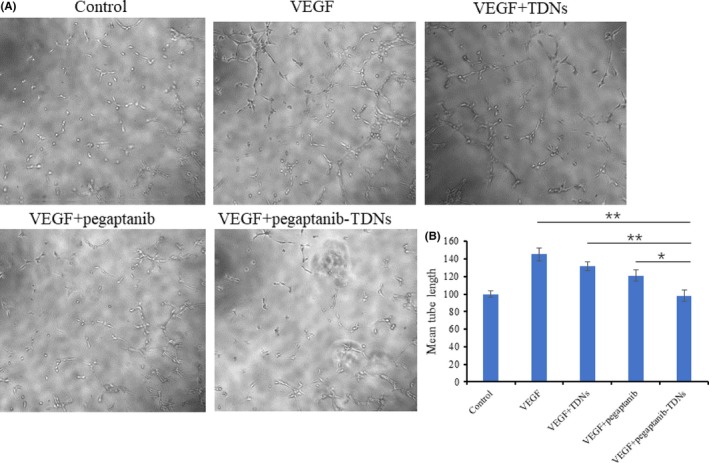
A, Pegaptanib‐TDNs inhibited the VEGF‐induced tube formation in HUVECs. B, Quantification of the mean tube length. Data are presented as mean ± SD (n = 3). Statistical analysis: *P < 0.05, **P < 0.01

Tube formation assay was carried out in order to test the influences of these materials on angiogenesis. Capillary tube formation induced by VEGF was reduced when HUVECs incubated with 375 nmol/L pegaptanib‐TDNs, but the inhibition effect was not obvious in the 375 nmol/L pegaptanib group (Figure 4A). Quantitative analysis of mean tube length was measured by ImageJ. As shown in Figure 4B, pegaptanib‐TDNs remarkably decreased the tube length (VEGF: 155 ± 7.07 μm; VEGF + TDNs: 132 ± 1.95 μm; VEGF + pegaptanib: 121 ± 6.36 μm; VEGF + pegaptanib‐TDNs: 98 ± 6.36 μm; VEGF + pegaptanib‐TDNs vs VEGF or VEGF + TDNs: *P* < 0.01; VEGF + pegaptanib‐TDNs vs VEGF + pegaptanib: *P* < 0.05).

These results suggested that pegaptanib loaded onto TDNs could effectively enhance the ability of pegaptanib to inhibit proliferation, migration and tube formation of HUVECs.

### 3.4 Cell binding capacity of pegaptanib and pegaptanib‐TDNS to HUVECS

**Figure 5:**
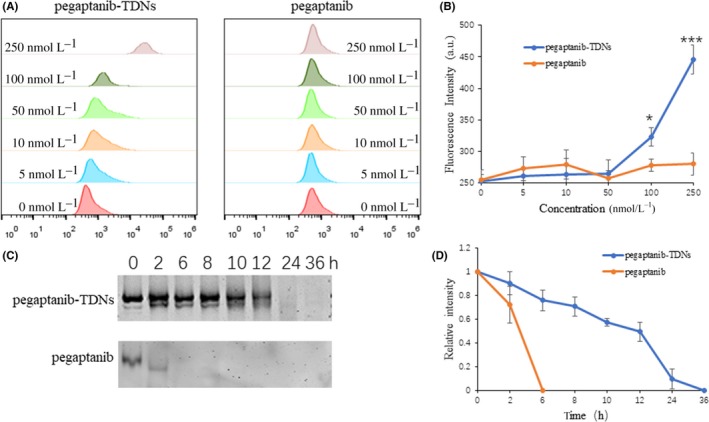
A, Pegaptanib‐TDNs and pegaptanib at a series of concentrations binding to HUVECs were measured by flow cytometry. B, Quantification of fluorescence intensity in different concentrations. C, The serum stability of pegaptanib‐TDNs and pegaptanib incubated with 10% FBS in 37°C. D, Quantification of the relative intensity at different time points. Data are presented as mean ± SD (n = 3). Statistical analysis: *P < 0.05, ***P < 0.001 (pegaptanib‐TDNs vs pegaptanib)

Pegaptanib and pegaptanib‐TDNs were labelled with cy5. Fluorescent signal was detected by flow cytometry to measure pegaptanib binding to HUVECs. When the concentration was below 50 nmol/L, the fluorescence signal of pegaptanib‐TDNs slowly increased, but with the increase of the concentration, especially at 250 nmol/L, the fluorescence signal obviously increased whereas the fluorescent signal was not observed any change in pegaptanib group (Figure 5A). Quantitative analysis of fluorescence intensity was shown in Figure 5B. The results suggested that TDNs conjugated with pegaptanib could enhance cell binding capacity of pegaptanib.

### 3.5 The serum stability of pegaptanib and pegaptanib‐TDNs

Subsequently, the serum stability of pegaptanib and pegaptanib‐TDNs was evaluated. The result of gel electrophoresis demonstrated that pegaptanib‐TDNs was almost completely degraded after incubated with 10% (*v*/*v*) FBS at 37°C in 5% CO_2_ for 24 hours, but pegaptanib occurred obvious degradation at 2 hours and complete degradation <6 hours (Figure 5C). Statistical analysis in Figure 5D showed that pegaptanib was degraded more rapidly than pegaptanib‐TDNs.

## 4 DISCUSSION

In this study, we synthesized pegaptanib‐TDNs and investigated the effect of anti‐tumour and anti‐angiogenesis. Pegaptanib, an RNA aptamer, was successfully linked to a vertex of TDN (Figure 1). Pegaptanib‐TDNs could inhibit proliferation of HUVECs and Cal27 in a concentration‐dependent manner. When these two cell lines were cultured with 375 nmol/L pegaptanib‐TDNs, a significant inhibition of cell proliferation was observed. However, pegaptanib had no apparent restraint on cell proliferation in the same concentration (Figure 2). Cal27 with VEGF high expression is a human tongue squamous cell carcinoma cell line.^38^ Abundant blood vessels provide essential conditions for its growth and metastasis.^39^ Some antibodies and aptamers binding to VEGF might interfere with notch signalling pathways,^40^ which finally cause cell inhibition. Pegaptanib‐TDNs could on the one hand inhibit HUVECs proliferation to cut off the nutritional supply to the tumour, and on the other hand, inhibit the proliferation of tumour cell itself. Then, we observed that pegaptanib‐TDNs could inhibit the proliferation, migration and tube formation of HUVECs induced by VEGF, while pegaptanib had no or just a little effect (Figures 3 and 4). The results demonstrated that antagonism of pegaptanib towards VEGF was increased when it was loaded onto TDNs. To explore the possible mechanism of the increased anti‐tumour and anti‐angiogenesis ability of pegaptanib‐TDNs, we evaluated the cell binding capacity to HUVECs and the serum stability of pegaptanib and pegaptanib‐TDNs. As shown in Figure 5, pegaptanib‐TDNs had stronger cell binding capacity and serum stability. When they were added into the cell media respectively, pegaptanib‐TDNs had enough time and quantity to bind to VEGF and make a difference in cell proliferation, migration and tube formation. It suggested that pegaptanib‐TDNs could circulate in the body as far as possible long time and resist to the degradation of various enzymes. These could enhance the anti‐angiogenesis and anti‐tumour activity of pegaptanib in vivo.

## 5 CONCLUSIONS

Taken together, we put forward a novel drug carrier for pegaptanib. TDNs could help pegaptanib overcome the limitations of aptamer and broaden its application in VEGF‐targeting cancer therapy. Our results demonstrated again that TDNs might be a vehicle of potential value for disease therapy.

## CONFLICT OF INTEREST

All authors declare no conflict of interest.

## Data Availability

The data that support the findings of this study are available from the corresponding author upon reasonable request.
